# Supplementary material for: Prospective Study Comparing Outcome following Complete Polypropylene Suture Ligation versus Partial Thin Film Band Attenuation of Congenital Portosystemic Shunts in Dogs
Source: Vet Sci. 2023 Jul 23;10(7):480. doi: 10.3390/vetsci10070480 (PMC10383284; doi:10.3390/vetsci10070480)
Supplement: Supplementary file 1 [file vetsci-10-00480-s001.zip › vetsci-2474627-supplementary.pdf]

Portosystemic Shunt Quality of Life Questionnaire: AFTER SURGERY

YOUR PET"S CLINICAL SIGNS AND QUALITY OF LIFE AFTER SURGERY

* Indicates required question

1. Email *

2. Please put your phone number below in case we need to contact you about this questionnaire. We collected your email above so that you can automatically be sent a copy your questionnaire responses to keep for future reference regarding your pet's shunt relate[illegible] quality of life signs. Please be assured that we will not use or distribute your email or phon[illegible] number for any other purpose.

3. What is your name? *

4. What is your pet's name?

5. Please add today's date below *

Example: January 7, 2019

6. How often does your pet have seizures? *

Mark only one oval.

- Never
- Less than once a month
- Monthly
- Weekly
- Daily

7. Please mark below how severely seizures impacts your pet's quality of life? *

Mark only one oval.

	0	1	2	3	4	5	6	7	8	9	10	
Not												Could not be worse

8. How often does your pet demonstrate 'head-pressing' behaviour? *

Mark only one oval.

- Never
- Less than once a month
- Monthly
- Weekly
- Daily

9. Please mark below how severely 'head-pressing' impacts your pet's quality of life? *

Mark only one oval.

	0	1	2	3	4	5	6	7	8	9	10	
Not												Could not be worse

10. How often does your pet demonstrate 'circling' behaviour? *

Mark only one oval.

- Never
- Less than once a month
- Monthly
- Weekly
- Daily

11. Please mark below how severely 'circling' impacts your pet's quality of life? *

Mark only one oval.

	0	1	2	3	4	5	6	7	8	9	10	
Not												Could not be worse

12. How often does your pet appear disorientated'? *

Mark only one oval.

- Never
- Less than once a month
- Monthly
- Weekly
- Daily

13. Please mark below how severely 'disorientation' impacts your pet's quality of life? *

Mark only one oval.

	0	1	2	3	4	5	6	7	8	9	10	
Not												Could not be worse

14. How often does your pet demonstrate aggressive behaviour? *

Mark only one oval.

- Never
- Less than once a month
- Monthly
- Weekly
- Daily

15. Please mark below how severely 'aggressive behaviour' impacts your pet's quality of life?

Mark only one oval.

	0	1	2	3	4	5	6	7	8	9	10	
Not												Could not be worse

16. How often does your pet 'collapse'? *

Mark only one oval.

- Never
- Less than once a month
- Monthly
- Weekly
- Daily

17. Please mark below how severely 'collapse' impacts your pet's quality of life? *

Mark only one oval.

	0	1	2	3	4	5	6	7	8	9	10	
Not												Could not be worse

18. How often does your pet demonstrate 'wobbliness/difficulty walking properly'? *

Mark only one oval.

- Never
- Less than once a month
- Monthly
- Weekly
- Daily

19. Please mark below how severely 'wobbliness/difficulty walking properly' impacts your pet's quality of life?

Mark only one oval.

	0	1	2	3	4	5	6	7	8	9	10	
Not	◯	◯	◯	◯	◯	◯	◯	◯	◯	◯	◯	Could not be worse

20. How often does your pet have 'unresponsive/vacant' episodes? *

Mark only one oval.

- Never
- Less than once a month
- Monthly
- Weekly
- Daily

21. Please mark below how severely 'unresponsive/vacant' episodes' impacts your pet's qualit of life?

Mark only one oval.

	0	1	2	3	4	5	6	7	8	9	10	
Not	◯	◯	◯	◯	◯	◯	◯	◯	◯	◯	◯	Could not be worse

22. How often does your pet demonstrate blindness? *

Mark only one oval.

- ◯ Never
- ◯ Less than once a month
- ◯ Monthly
- ◯ Weekly
- ◯ Daily

23. Please mark below how severely blindness impacts your pet's quality of life? *

Mark only one oval.

	0	1	2	3	4	5	6	7	8	9	10	
Not	◯	◯	◯	◯	◯	◯	◯	◯	◯	◯	◯	Could not be worse

24. How often does your pet demonstrate excessive salivation (dribbling of saliva)? *

Mark only one oval.

- ◯ Never
- ◯ Less than once a month
- ◯ Monthly
- ◯ Weekly
- ◯ Daily

25. Please mark below how severely excessive salivation impacts your pet's quality of life? *

Mark only one oval.

	0	1	2	3	4	5	6	7	8	9	10	
Not	◯	◯	◯	◯	◯	◯	◯	◯	◯	◯	◯	Could not be worse

26. How often does your pet demonstrate lethargy/weakness? *

Mark only one oval.

- ◯ Never
- ◯ Less than once a month
- ◯ Monthly
- ◯ Weekly
- ◯ Daily

27. Please mark below how severely lethargy/weakness impacts your pet's quality of life? *

Mark only one oval.

	0	1	2	3	4	5	6	7	8	9	10	
Not	◯	◯	◯	◯	◯	◯	◯	◯	◯	◯	◯	Could not be worse

28. How often does your pet vomit? *

Mark only one oval.

- ◯ Never
- ◯ Less than once a month
- ◯ Monthly
- ◯ Weekly
- ◯ Daily

29. Please mark below how severely vomiting impacts your pet's quality of life? *

Mark only one oval.

	0	1	2	3	4	5	6	7	8	9	10	
Not	◯	◯	◯	◯	◯	◯	◯	◯	◯	◯	◯	Could not be worse

30. How often does your pet have diarrhoea? *

Mark only one oval.

- ◯ Never
- ◯ Less than once a month
- ◯ Monthly
- ◯ Weekly
- ◯ Daily

31. Please mark below how severely diarrhoea impacts your pet's quality of life? *

Mark only one oval.

	0	1	2	3	4	5	6	7	8	9	10	
Not	◯	◯	◯	◯	◯	◯	◯	◯	◯	◯	◯	Could not be worse

32. How often does your pet have inappetance? *

Mark only one oval.

- ◯ Never
- ◯ Less than once a month
- ◯ Monthly
- ◯ Weekly
- ◯ Daily

33. Please mark below how severely inappetance impacts your pet's quality of life? *

Mark only one oval.

	0	1	2	3	4	5	6	7	8	9	10	
Not	◯	◯	◯	◯	◯	◯	◯	◯	◯	◯	◯	Could not be worse

34. Drinking: do you consider the amount of water your pet drinks per day to be: *

Mark only one oval.

- ◯ Normal
- ◯ Slightly more than expected
- ◯ Definitely excessive
- ◯ Severely excessive
- ◯ Don't know

35. Please mark below how severely drinking frequency impacts your pet's quality of life? *

Mark only one oval.

	0	1	2	3	4	5	6	7	8	9	10	
Not	◯	◯	◯	◯	◯	◯	◯	◯	◯	◯	◯	Could not be worse

36. Urination: do you consider the amount of urination your pet performs per day to be: *

Mark only one oval.

- ◯ Normal
- ◯ Slightly more than expected
- ◯ Definitely excessive
- ◯ Severely excessive
- ◯ Don't know

37. Please mark below how severely urination frequency impacts your pet's quality of life? *

Mark only one oval.

	0	1	2	3	4	5	6	7	8	9	10	
Not	◯	◯	◯	◯	◯	◯	◯	◯	◯	◯	◯	Could not be worse

38. Does your pet have difficulty urinating? *

Mark only one oval.

- ◯ Never
- ◯ Less than once a month
- ◯ Monthly
- ◯ Weekly
- ◯ Daily

39. Please mark below how severely 'difficulty urinating' impacts your pet's quality of life? *

Mark only one oval.

	0	1	2	3	4	5	6	7	8	9	10	
Not	◯	◯	◯	◯	◯	◯	◯	◯	◯	◯	◯	Could not be worse

40. Do you see blood in your pet's urine? *

Mark only one oval.

- ◯ Never
- ◯ Less than once a month
- ◯ Monthly
- ◯ Weekly
- ◯ Daily

41. Please mark below how severely having 'blood in urine' impacts your pet's quality of life? *

Mark only one oval.

	0	1	2	3	4	5	6	7	8	9	10	
Not	◯	◯	◯	◯	◯	◯	◯	◯	◯	◯	◯	Could not be worse

42. Has your pet suffered from bladder stones or urinary obstruction SINCE SURGERY? If yes, please give details below

____

____

____

____

____

43. Please mark below how severely having bladder stones and/or urinary obstruction impacts your pet's quality of life?

Mark only one oval.

	0	1	2	3	4	5	6	7	8	9	10	
Not	◯	◯	◯	◯	◯	◯	◯	◯	◯	◯	◯	Could not be worse

44. Would you consider your pet to be small/underweight for their breed and age now? *

Mark only one oval.

- ◯ Yes
- ◯ No
- ◯ Not sure

45. How active is your pet? *

Mark only one oval.

	0	1	2	3	4	5	6	7	8	9	10	
Not	◯	◯	◯	◯	◯	◯	◯	◯	◯	◯	◯	Could not be more active

46. How willing is your pet to play? *

Mark only one oval.

	0	1	2	3	4	5	6	7	8	9	10	
Not	◯	◯	◯	◯	◯	◯	◯	◯	◯	◯	◯	Could not be more willing

47. How willing is your pet to interact with yourselves? *

Mark only one oval.

	0	1	2	3	4	5	6	7	8	9	10	
Not	◯	◯	◯	◯	◯	◯	◯	◯	◯	◯	◯	Could not be more willing

48. How willing is your pet to exercise? *

Mark only one oval.

	0	1	2	3	4	5	6	7	8	9	10	
Not	◯	◯	◯	◯	◯	◯	◯	◯	◯	◯	◯	Could not be more willing

49. How willing is your pet to interact with other dogs or cats (as applicable)? *

Mark only one oval.

	0	1	2	3	4	5	6	7	8	9	10	
Not	◯	◯	◯	◯	◯	◯	◯	◯	◯	◯	◯	Could not be more willing

50. If any signs persisted after surgery, how long did they last for? *

Mark only one oval.

- ◯ < 4 weeks
- ◯ 1-6 months
- ◯ > 6 months
- ◯ Signs are still present and ongoing

51. Has your pet has any relapse in signs related to the shunt condition? If yes, please detail below:

____

____

____

____

____

52. Please tick all medications/special diet that your pet is receiving below *

Check all that apply.

- ☐ Lactulose (liquid laxative)
- ☐ Antibiotics (any type)
- ☐ Special diet/liver diet
- ☐ levetiracetam (anti-seizure drug)
- ☐ phenobarbitone (anti-seizure drug)
- ☐ No medications or special diet
- ☐ Other: _______________________________

53. Please state your pet's diet below: *

____

54. Do you feel your pet has improved in body condition since surgery? *

Mark only one oval.

	0	1	2	3	4	5	6	7	8	9	10	
Not	◯	◯	◯	◯	◯	◯	◯	◯	◯	◯	◯	Could not have improved more

55. How improved is your pet since surgery generally? *

Mark only one oval.

	0	1	2	3	4	5	6	7	8	9	10	
Not	◯	◯	◯	◯	◯	◯	◯	◯	◯	◯	◯	Could not have improved more

56. How satisfied are you with your pet's response to surgery? *

Mark only one oval.

	0	1	2	3	4	5	6	7	8	9	10	
Not	◯	◯	◯	◯	◯	◯	◯	◯	◯	◯	◯	Could not have improved more

57. Please rate your pet's overall quality of life PRIOR TO SHUNT DIAGNOSIS below: *

Mark only one oval.

	0	1	2	3	4	5	6	7	8	9	10	
Wor	◯	◯	◯	◯	◯	◯	◯	◯	◯	◯	◯	Best imaginable

58. Please rate your pet's overall quality of life NOW: *

Mark only one oval.

	0	1	2	3	4	5	6	7	8	9	10	
Wor	◯	◯	◯	◯	◯	◯	◯	◯	◯	◯	◯	Best imaginable

59. Thank you very much for taking the time to fill out this questionnaire. If you have any furth queries or concerns relating to your pet's shunt after filling out this questionnaire and/or would like us to contact you to discuss these, please give details below:

____

____

____

____

____

60. Would you be happy to fill out this questionnaire again in the future to monitor progression of your pet's quality of life following shunt treatment

Mark only one oval.

◯ YES

◯ NO

This content is neither created nor endorsed by Google.

Google Forms
